# Revival of Cancer Registration in Karachi, Pakistan

**DOI:** 10.31557/APJCP.2021.22.1.1

**Published:** 2021-01

**Authors:** Muhammad Asif Qureshi

**Affiliations:** *Dow International* *Medical* *College, Dow University of Health Sciences, Karachi, Pakistan. *

**Keywords:** Cancer registry, cancers in Karachi-Pakistan, Karachi cancer registry, dow cancer registry

**Dear Editor**

Effective national level cancer registration system does not exist in Pakistan (Qureshi et al., 2015). It has always been a matter of concern for all stakeholders within the country as well as amongst relevant international authorities. Unfortunately, no minimal and inefficacious efforts have been made in the past by the policy makers in Pakistan to facilitate the establishment of a nation-wide functional cancer registration authority. However, regional cancer registries have substantially contributed towards establishing cancer statistics in Pakistan. One such regional cancer registry was the Karachi Cancer Registry (KCR) which was founded by Dr. Yasmin Bhurgri and published several reports detailing cancer patterns from Karachi South. However, after the death of Dr. Yasmin, the KCR went practically nonfunctional until recently when it was revived by a group of relevant stakeholders from various institutions in Karachi.

Recent publication of a number of reports detailing cancer patterns in Karachi is noticeable and effectively highlight revival of cancer registration in Karachi, Pakistan. Qureshi et al in 2016 reported 6-year (2010-2015) cancer data from the Dow Cancer Registry which represents the largest public sector diagnostic and reference laboratory of the city (Qureshi et al.,2016). More recently, another report from the Dow Cancer Registry detailed cancer patterns in Karachi during 2010-2019 (Qureshi et al., 2020). Importantly, revival of the KCR was announced in a recent report which describes cancer patterns in Karachi during 2017-2019 (Pervez et al., 2020). It is important to mention there that after Dr. Bhurgri’s death, this is the first notable report from the platform of the KCR and thus a considerable step towards establishing cancer registration in Karachi. These regular publications describing high quality regional cancer data indicate the commitment and dedication demonstrated by cancer researchers from the largest city of Pakistan towards establishing cancer registration in the city.

While these regional efforts are highly appreciable, it is now high time that an effective national cancer registration system is formulated in Pakistan. The government of Pakistan, relevant policy makers as well as international cancer registration authorities will have to play a vital role in this regard. 

## Conflict of interest

Author (Prof. Dr. M. Asif Qureshi) declares that he is founding in charge of the Dow Cancer Registry and a contributing member towards the Karachi Cancer Registry.
